# Mapping elemental solutes at sub-picogram levels during aqueous corrosion of Al alloys using diffusive gradients in thin films (DGT) with LA-ICP-MS

**DOI:** 10.1007/s00216-024-05288-8

**Published:** 2024-04-16

**Authors:** Gulnaz Mukhametzianova, Stefan Wagner, Magdalena Eskinja, Masoud Moshtaghi, Gregor Mori, Thomas Prohaska

**Affiliations:** 1 Department of General, Analytical and Physical Chemistry, Chair of General and Analytical Chemistry, Montanuniversität Leoben, Franz-Josef-Strasse 18, 8700 Leoben, Austria; 2 Christian Doppler Laboratory for Inclusion Metallurgy in Advanced Steelmaking, Franz-Josef-Strasse 18, 8700 Montanuniversität Leoben, Leoben, Austria; 3 Laboratory of Steel Structures, LUT University, P.O. Box 20, 53851 Lappeenranta, Finland

**Keywords:** Passive sampling, Aluminium, Corrosion, Chemical imaging, Diffusion

## Abstract

A novel approach using diffusive gradients in thin films (DGT) with laser ablation inductively coupled plasma mass spectrometry (LA-ICP-MS) for two-dimensional mapping of elemental solute release at sub-picogram levels during aqueous corrosion of Al alloys is presented. Evaluation of different DGT gels with mixed micro-sized binding phases (polyacrylamide-Chelex-Metsorb, polyurethane (PU)-Chelex-Metsorb, PU-Chelex-Zr(OH)_4_) demonstrated the superior performance of PU gels due to their tear-proof handling, low shrinkage, and compliance with green chemistry. DGT devices containing PU-Chelex-Zr(OH)_4_ gels, which have not been characterized for Al sampling before, showed quantitative uptake of Al, Zn, and Cu solutes over time (*t* = 4–48 h) with higher Al capacity (*Γ*_DGT_ = 6.25 µg cm^−2^) than different gels. Application of PU-Chelex-Zr(OH)_4_ gels on a high-strength Al-Cu alloy (Al2219) exposed to NaCl (*w* = 1.5%, pH = 4.5, *T* = 21 °C) for 15 min in a novel piston-type configuration revealed reproducible patterns of Al and Zn co-solubilization with a spatial expansion ranging between 50 and 1000 µm. This observation, together with complementary solid-state data from secondary electron microscopy with energy-dispersive X-ray spectroscopy, showed the presence of localized pitting corrosion at the material surface. Detection limits for total solute masses of Al, Zn, and Cu were ≤0.72 pg, ≤8.38 pg, and ≤0.12 pg, respectively, for an area of 0.01 mm^2^, demonstrating the method’s unique capability to localize and quantify corrosion processes at ultra-trace levels and high resolution. Our study advances the assessment of Al alloy degradation in aqueous environments, supporting the design of corrosion-resistant materials for fostering technological safety and sustainability.

**Graphical Abstract:**

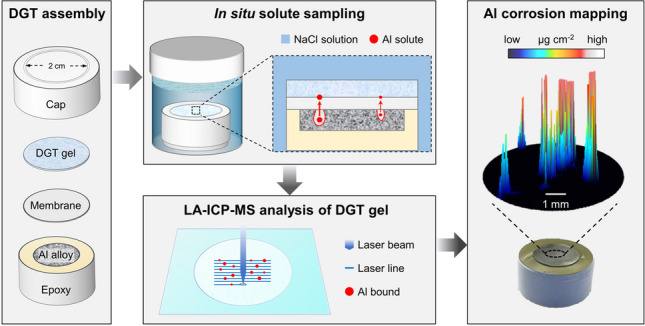

**Supplementary Information:**

The online version contains supplementary material available at 10.1007/s00216-024-05288-8.

## Introduction

Aluminum (Al) alloys are extensively used in automotive, naval, and aircraft manufacturing [1–4]. Given their high strength-to-weight ratio, infinite recyclability, and energy-efficient fabrication when produced from scrap, they are essential building blocks towards sustainable transportation and circular metallurgy [5–7]. Besides, Al alloys are attractive for their high corrosion resistance, owing to a dense passivated oxide film formed on their surface, preventing uniform degradation for enhanced material longevity [8]. However, Al alloys, as other metallic alloys, suffer from localized corrosion when exposed to aqueous environments [9, 10]. Pitting corrosion is of particular relevance for causing damage of Al alloys [11], which is both costly and dangerous given their delicate application fields [12, 13]. The general reaction of Al corrosion in aqueous solutions is presented in Eqs. 1–3 [10]:

1 $${\text{Al}}\rightleftharpoons {{\text{Al}}}^{3+}+3{{\text{e}}}^{-}$$

2 $$2{{\text{H}}}_{2}{\text{O}}+{{\text{O}}}_{2}+4{{\text{e}}}^{-}\rightleftharpoons {4{\text{OH}}}^{-}$$

3 $${{\text{Al}}}^{3+}+{3{\text{OH}}}^{-}\rightleftharpoons {{\text{Al}}({\text{OH}})}_{3}$$

The anodic oxidation leads to Al^3+^ release (Eq. 1) and cathodic reduction of dissolved oxygen leads to production of hydroxide ions (Eq. 2), modulating pH and eventually forming Al(OH)_3_ at the alloy surface (Eq. 3). Pitting corrosion is due to localized depassivation of the oxide surface film, which can be initiated by chemical heterogeneities such as intermetallic particles causing electrochemical potential differences in the alloy matrix [14, 15]. Subsequent oxidation of the underlying Al results in formation of irregularly shaped pits with diameters and depths ranging from nanometer to low millimeter scales [16, 17].

To better understand the localized reaction mechanisms involved in aqueous Al corrosion, extensive research based on surface-sensitive ex situ analysis techniques has been conducted. For instance, scanning electron microscopy with energy-dispersive x-ray spectroscopy (SEM–EDX), X-ray photoelectron spectroscopy (XPS), and time of flight secondary ion mass spectrometry (ToF–SIMS) have been applied [18–21]. However, assessing the distribution of element-specific dissolution effects and their rates of interaction in situ, i.e., while corrosion takes place, remains elusive. The only technique previously used for this purpose was in situ scanning Kelvin probe force microscopy (SKPFM) [22], which is a high-resolution electrochemical potential measurement method. Besides its nanoscale capabilities, SKPFM provides no elemental information and is challenged by probe calibration and matrix interferences [23], limiting data reproducibility and comparability. Thus, there is currently no technique available by which the locally solubilized mass of Al and other elements can be directly quantified at adequate spatial scales, especially at the initial stages of corrosion, when only ultra-trace levels of solutes are released from isolated sites across the material surface. Therefore, new approaches capable of simultaneous, selective, and quantitative in situ mapping of multiple solute corrosion products at high spatial resolution and with low limits of detection are needed.

Diffusive gradients in thin films (DGT) is a passive sampling technique widely used in environmental research to assess elemental solubilization processes in waters, sediments, and soils. Since its first development in 1994 [24], DGT has been rapidly advanced from bulk sampling of solutes towards mapping applications at high spatial resolution [25, 26]. The technique uses thin hydrogels containing specific binding phases with high selectivity and capacity for the target analyte(s). For example, Chelex, an iminodiacetate (IDA)-based chelating resin, is typically applied for sampling metal cations, whereas Metsorb, a titanium dioxide-based adsorbent, and zirconium hydroxide (Zr(OH)_4_) are typical binding phases for (oxy)anions [27]. Polyacrylamide (PA), cross-linked using an agarose derivative, is the most common choice as a gel matrix, but recent studies indicated the superior performance of polyurethane (PU), an ether-based urethane polymer, due to its tear-proof properties [28, 29]. During sampling, the gel is applied to the sample surface under study and binds labile solute fractions in situ upon diffusive uptake. Subsequent chemical analysis of the gel surface by laser ablation inductively coupled plasma mass spectrometry (LA-ICP-MS) enables two-dimensional (2D) mapping of the sampled solute distribution at low detection limits (sub µg g^−1^) and high spatial resolution (< 100 µm) [30, 31]. A major analytical asset of combining DGT with LA-ICP-MS is the possibility to use matrix-matched gel standards with known analyte mass loadings for quantification [26]. The use of the gel’s major matrix element, carbon, as an internal normalization standard further allows to correct for fluctuations in the signal intensity originating from changes in aerosol formation, transportation, and ionization, which are major challenges in LA-ICP-MS analysis [32].

Recently, the first use of the distinct analytical advantages of DGT LA-ICP-MS in the field of corrosion research was presented [33]. That study was applied on a magnesium (Mg) alloy used in biomedical technologies and provided a full metrological validation of the technique, demonstrating its expansive potential to visualize localized corrosion processes in aqueous environments, which might extend to Al alloys as well. For Al sampling by DGT, previous work using conventional PA-Chelex gels for assessing dissolved Al concentrations in environmental waters showed that Al uptake by this gel type is negligible at pH > 8 [34, 35]. The reason is that Al speciation in solution is controlled by pH [36], with cationic Al^3+^, along minor amounts of AlOH^2+^ and Al(OH)_2_^+^, dominating at pH < 6.0, and anionic Al(OH)_4_^−^ dominating at pH > 7.5 [37], which is not taken up by the negatively charged IDA binding sites of Chelex. Although the selectivity of Chelex effectively enables fractionation of Al species, it hinders the assessment of total Al solute fractions released during aqueous corrosion where pH and hence Al speciation are modulated dynamically. Consequently, a mixed binding gel containing both Chelex and an anionic binding agent such as Metsorb or Zr(OH)_4_ would be beneficial for sampling total dissolved Al without species-dependent fractionation effects.

The aim of this work was to develop and evaluate a new DGT LA-ICP-MS approach capable of 2D, high-resolution mapping of elemental solubilization and diffusion, focusing on the release of total Al solute fractions during corrosion of Al alloys exposed to sodium chloride (NaCl) solution. The method development was based on two consecutive steps, including (1) assessment of performance characteristics of three different DGT gels containing mixed micro-sized binding phases for Al sampling and (2) evaluation of the effects of DGT deployment conditions on Al mapping. Finally, the best DGT configuration was applied on a technologically relevant Al-Cu alloy along with complementary solid-state imaging techniques to demonstrate the potential of the novel approach.

## Materials and methods

### Experimental design

An overview of the experimental design is provided in Table 1. In summary, three different types of mixed micro-sized binding phase DGT gels (named mixed binding gels throughout this paper) were applied to two different types of Al materials exposed to NaCl solution in four different experiments. Prior to their application, the performance of the gels was evaluated with respect to their mechanical properties, shrinkage effects, and analyte binding characteristics. Moreover, a novel piston-type deployment configuration for standardized DGT deployment in corrosion research was developed and compared to a typical tape-type deployment configuration applied in a preliminary experiment (experiment 0). In experiments 1–3, two different gels with differing gel matrices but the same binding phases were included, while three different gel deployment times were applied to investigate the effect of this parameter on quantitative Al solute mapping. Based on the results of experiments 1–3, an optimized approach was applied in experiment 4, which was repeated twice on different days to test the method reproducibility, while also applying optical microscopy and SEM–EDX on the used Al material to obtain complementary solid-state data.

**Table 1:** Summary of the experimental design and deployment conditions (details on DGT gels, Al materials, deployment configurations, and deployment times are provided in the following sections)

Experiment	DGT gel	Al material	Deployment configuration	Deployment time
0	PA-C	Al99.5	tape	24 h, 72 h
1	PA-C-M	Al99.5	piston	24 h, 72 h
2	PU-C-M	Al99.5	piston	24 h, 72 h
3	PA-C-M	Al2219	piston	80 min
4	PU-C-Zr	Al2219	piston	15 min

### Laboratory procedures, reagents, and solutions

Laboratory water type 1 (*ρ* = 18.2 MΩ × cm at 25 °C) was obtained from a Milli-Q device (Merck Millipore, DE). High-purity nitric acid (HNO_3_) was obtained by sub-boiling analytical grade HNO_3_ (*w* = 65%, p.a., Chem-Lab, BE) in perfluoroalkoxy-polymer units (DST-1000 and DST-4000, Savillex, USA). Acrylonitrile butadiene styrene DGT devices (R-SDU; DGT Research Ltd., UK), glass materials, polytetrafluoroethylene (PTFE) spacers, and other plastic consumables used for DGT preparation, deployment, and analysis were soaked in sub-boiled HNO_3_ (*w* = 5–10%) for at least 24 h, rinsed with laboratory water type 1, and then dried in a laminar flow module (Spetec GmbH, DE). Acrylamide solution (*w* = 40%, Sigma-Aldrich, AT), cross-linker (*w* = 2%, DGT Research Ltd., UK), ammonium peroxodisulfate (APS; ≥ 98.0%, p.a., VWR, AT), N,N,N′,N′-tetramethylethylenediamine (TEMED; ~ 99%, BioReagent, suitable for electrophoresis, Merck, DE), ethanol absolute (≥ 99.8%, VWR, AT), and laboratory water type 1 were used for DGT gel preparation. The aqueous NaCl solution was prepared from sodium chloride (≥ 99.5%, p.a., Roth, AT) diluted in laboratory water type 1. Single-element stock solutions were prepared by dissolving aluminum chloride anhydrous (AlCl_3_; ≥ 99%, VWR), zinc chloride anhydrous (ZnCl_2_; ≥ 99.95%, VWR), and copper(II) chloride dihydrate (CuCl_2_ × 2H_2_O; ≥ 99%, VWR). These stocks were spiked to the aqueous NaCl solution to prepare the immersion solutions for the assessment of analyte binding characteristics of the different DGT gels. If necessary, pH adjustments were carried out using aqueous NaOH (*c* = 1 mol l^−1^) prepared from sodium hydroxide monohydrate (NaOH × 1H_2_O; ≥ 99.996%, metals basis, Thermo Scientific). Hydrogen peroxide (H_2_O_2_; *w* = 30%, Merck, DE) and sub-boiled HNO_3_ (*w* = 65%) were used for microwave-assisted acid digestion. Ethanol (*w* = 95.6%) or ethanol absolute (≥ 99.8%; both VWR) and deionized water were used for cleaning Al samples after polishing. An analytical balance (BL224 BASIC, XS instruments, IT) with a readability of 0.0003 g was used for weighing. Cleaning of consumables, preparation of DGT gels, immersion solutions, and standards, as well as ICP-MS analyses, were performed in a clean room laboratory (ISO class 8).

### Sample materials and preparation

Two types of Al materials were used. The first was a commercial-grade Al material (Al99.5) with a thickness of 1.95 mm. The second was a cold-rolled high-strength Al-Cu alloy (Al2219) [14, 38] with a thickness of 0.70 mm. The bulk elemental composition of the samples was determined using spark optical emission spectroscopy (ARL iSpark, Thermo Fisher Scientific, DE) and is shown in Table 2.

**Table 2:** Elemental composition of the Al99.5 and Al2219 materials

Material	Element, *w* (%)								
	Mg	Si	V	Mn	Fe	Cu	Zn	Zr	Al
Al99.5	< 0.01	0.05	0.01	< 0.01	0.32	< 0.01	< 0.01	< 0.01	Balance
Al2219	0.12	0.04	0.07	0.23	0.11	5.8	0.03	0.03	Balance

For sample preparation, Al materials were cut, ground, and polished. For experiment 0, two Al99.5 samples were cut to rectangular shapes of about 5 cm × 6.5 cm, whereas for experiments 1–4, four Al99.5 and three Al2219 samples were cut into circular shapes (*d* = 18 mm) and mounted into epoxy (EpoFix Kit, Struers, AT) cylinders (*d* = 25 mm,* h* = 12 mm). To compare the effect of different metallographic procedures on the material surface roughness, two different approaches were applied. In the first approach, the Al samples (six Al99.5 and one Al2219) were ground using silicon carbide (SiC) grinding paper (grain size P240, P500, P1200, P2000, P2500) and then polished using 9-µm, 3-µm, and finally 1-µm diamond suspensions. In the second approach, the Al samples (two Al2219) were ground using SiC grinding paper (grain size P180, P320, P600, P1200) and then polished using a 3-µm diamond suspension and water-based lubricant on a medium hard polishing wool cloth (MD-mol, Struers, AT). Finally, polishing was finished in an additional step using chemical-resistant cloth (MD Chem, Struers, AT) with colloidal silica suspension (OP-U, Struers, AT). Polished samples were rinsed using ethanol and water, dried, and then stored either at room temperature (Al99.5) or at 6 °C in the fridge (Al2219) until use. Surface roughness measurements on the metallographic sections were performed using 3D optical microscopy (Alicona Infinite Focus G4, Alicona Imaging GmbH, AT).

### Preparation of DGT gels

Three different mixed binding gels were prepared, including polyacrylamide-Chelex-Metsorb (PA-C-M), polyurethane-Chelex-Metsorb (PU-C-M), and polyurethane-Chelex-Zr(OH)_4_ (PU-C-Zr). In addition, a conventional polyacrylamide-Chelex gel (PA-C) was prepared according to published procedures [34]. For all, a 0.1 g ml^−1^ suspension of milled Chelex (Chelex-100, sodium form, original particle size 37–75 µm, Sigma-Aldrich, AT) in laboratory water type 1 was used. Milling of Chelex to a particle size ≤ 10 µm and preparation of the suspension was were out as described elsewhere [29]. Accordingly, a 0.1 g ml^−1^ suspension of Metsorb with a particle size of 5 µm (HMRP5, Graver Technologies, USA) was prepared and used in PU-C-M gels.

Preparation of PA-C-M gels was carried out as detailed in Panther et al. [39] with minor modifications: An amount of 0.2 g Metsorb (HMRP5) and 0.5 ml of laboratory water type 1 was added to 1.5 ml PA stock solution mixed with 1 ml Chelex suspension. This mixture was sonicated for 10 min, then shaken by hand, and finally fixed in an overhead shaker at 5 rpm for at least 2 h. Afterwards, 15 µl of APS and 5 µl of TEMED were added, and the gel solution was pipetted between two glass plates separated by a PTFE spacer with a thickness of 0.25 mm. The gels were set at 45 °C for 45 min, removed from the glass plates, and hydrated in laboratory water type 1 for 24 h with three consecutive changes of water.

Preparation of PU-C-M and PU-C-Zr gels was carried out as detailed in Doolette et al. [29] and Kreuzeder et al. [28], respectively, with minor modifications: For PU-C-M, a volume of 1.5 ml Metsorb suspension was mixed with 1.5 ml Chelex suspension and filled up to 15 ml volume with PU stock solution consisting of HydroMed D4 (Advan Source Biomaterials, USA) dissolved in an ethanol-laboratory water type 1 mixture [28]. For PU-C-Zr, 15 g Zr(OH)_4_ precipitate, prepared from ZrOCl_2_ × xH_2_O (≥ 99.9%, metals basis; Thermo Scientific), was filled up to 100 ml volume with PU stock solution. From this, 13.5 ml was taken and mixed with 1.5 ml of Chelex suspension on an overhead shaker at 5 rpm for 2 h. Both PU gels were prepared using an automated coating device (byko-drive G, BYK, DE), enabling the fabrication of large PU gels (up to approximately 20 cm × 15 cm) with improved reproducibility as compared to manual coating. All gels were pre-conditioned in ~ 30 ml NaCl solution (*w* = 1.5%, pH = 4.5) overnight prior to their deployment on Al materials.

### Performance of DGT gels for Al mapping

The performance of the three mixed binding gels for Al mapping was assessed by evaluating their mechanical properties, potential shrinkage effects, and analyte binding characteristics. PA-C gels were also included in the evaluation of mechanical properties and shrinkage, but not binding characteristics given their well-documented pH-dependent Al uptake efficiency [34, 35].

Shrinkage of the gels was evaluated both after immersion in NaCl solution for 24 h and after vacuum-assisted drying for subsequent LA-ICP-MS analysis. The gels (*n* = 5) were cut to discs (*d* = 25 mm) and two replicates of each gel type were immersed in 30 ml NaCl (*w* = 1.5%, pH = 4.5, *T* = 21 °C) reflecting the pre-conditioning process. The other two replicates were immersed in 30 ml of laboratory water type 1 as control. The vials were fixed in a horizontal shaker set to 160 rpm. After 24 h, all gels were recovered from the solutions and their area was measured using a digital caliper. Subsequently, DGT gels were fixed on a polyethersulfone (PES) membrane (pore size 0.45 µm; Supor 450, Pall Corporation, USA) and fully desiccated in a vacuum gel dryer (UNIGELDRYER 3545, Uniequip, DE). Finally, the gel area after drying was measured using a caliper and the relative shrinkage (in %) as compared to the control group was calculated. Moreover, an additional set of gel discs (*n* = 3) was weighed before and after drying to determine the gravimetric water content of the different gel types.

Evaluation of the gel analyte binding characteristics was based on mass balance and DGT bulk sampling experiments [27]. For PA-C-M and PU-C-M gels, individual gel discs (*n* = 4) were deployed for 24 h in 10 ml of one of two well-shaken NaCl solutions (*w* = 1.5%, pH = 4.5,* T* = 22 °C) ranging in Al concentration from 1213 µg l^−1^ ± 6 µg l^−1^ to 4960 µg l^−1^ ± 9 µg l^−1^. Four PA-C-M and PU-C-M gels each were included as blanks, i.e., not deployed in the immersion solution. After 24 h, the gels were retrieved from the immersion solution and three gel replicates were eluted for 24 h in 5 ml HNO_3_ (*c* = 5 mol l^−1^). To calculate the Al uptake and elution efficiency, the immersion solutions before and after gel deployment, as well as the gel eluates, were analyzed by ICP-MS (NexION 5000, PerkinElmer, USA). The remaining gel replicate per gel type was dried as stated above and mounted on a glass plate following published procedures [30] to serve as gel standard for LA-ICP-MS calibration of PA-C-M and PU-C-M gel samples.

For PU-C-Zr gels, a bulk sampling experiment was conducted for evaluating linear mass accumulation of analytes over time. The PU-C-Zr gel discs were placed in DGT piston devices and overlain by a 0.8-mm-thick agarose cross-linked PA diffusive layer [30] and a 10-µm-thick polycarbonate membrane (pore size 0.2 μm; Whatman Nuclepore, UK). A 0.4-mm-thick spacer was placed underneath the PU-C-Zr gel to compensate for the lower thickness as compared to common PA-based binding gels. The DGT devices (*n* = 4) were deployed for 4, 8, 12, 18, 24, 30, 36, and 48 h in 3.5 l of a well-stirred NaCl solution (*w* = 1.5%, pH = 4.5, *T* = 23 °C) containing 892 µg l^−1^ ± 23 µg l^−1^ of Al, 80 µg l^−1^ ± 2 µg l^−1^ of Zn, and 86 µg l^−1^ ± 3 µg l^−1^ of Cu. Four devices were included as blanks. After each deployment time, four devices were retrieved from the immersion solution and disassembled. To assess the time-dependent uptake of Al, Zn, and Cu by PU-C-Zr gels, three gel replicates per time point were digested using 5 ml HNO_3_ (*w* = 65%) and 1 ml H_2_O_2_ (*w* = 30%) in a microwave (Multiwave PRO, Anton Paar, AT) according to Kreuzeder et al. [28] and analyzed by ICP-MS (NexION 2000B, PerkinElmer, USA). Diffusion coefficients used to calculate the theoretical mass uptake were 4.28 × 10^−6^ cm^2^ s^−1^ for Al [40], 5.76 × 10^−6^ cm^2^ s^−1^ for Zn, and 5.90 × 10^−6^ cm^2^ s^−1^ for Cu at 25 °C [41]. As for PA-C-M and PU-C-M gels, the remaining gel replicate per time point was prepared as a gel standard for LA-ICP-MS calibration of PU-C-Zr gel samples.

### DGT deployment on Al samples

**Fig. 1:**
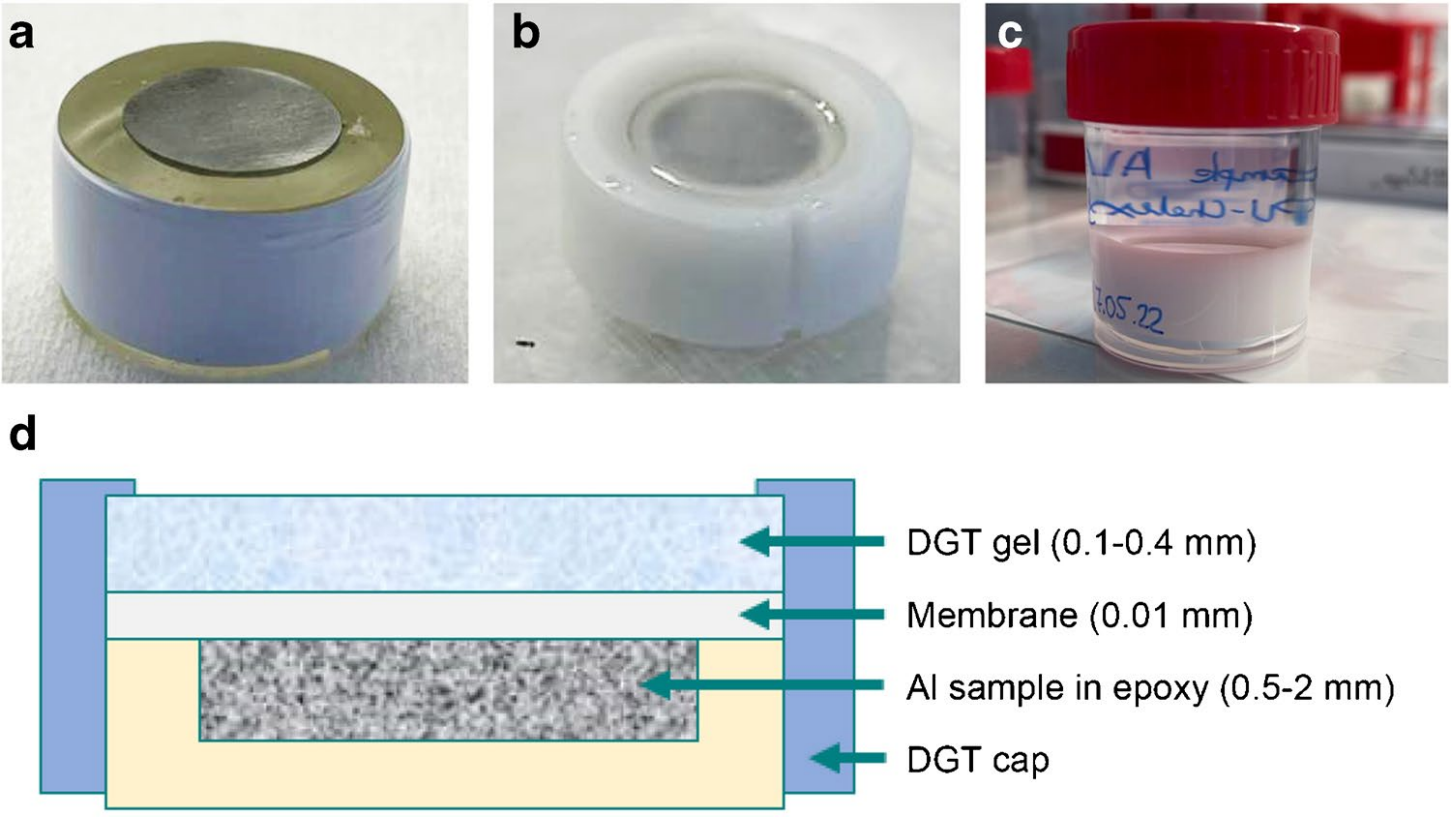
Piston-type deployment configuration for in situ sampling of localized Al release during aqueous corrosion, including Al sample mounted into epoxy (**a**), deployment assembly before (**b**) and during (**c**) immersion in NaCl solution and gel deployment, as well as cross-section through the deployment assembly showing the individual components and their approximate thickness (**d**; not to scale)

A novel piston-type deployment configuration (Fig. 1) was developed to deploy DGT binding gels on Al samples for in situ sampling of localized Al release. Polished Al samples mounted into epoxy were overlain by a polycarbonate membrane (thickness 10 µm, pore size 0.2 µm; Whatman Nuclepore, UK), followed by a DGT gel, which was fixed with a conventional DGT sampler cap (*d*_exposure window_ = 20 mm) fitting onto the epoxy cylinder. During the deployment period, which lasted from 15 min up to 72 h, the setup was placed into a 30-ml polypropylene vial filled with 20 ml NaCl solution (*w* = 1.5%, pH = 4.5), ensuring rapid initiation of Al corrosion [14]. After deployment, the DGT gels were rinsed with water, dried, and mounted on glass plates as stated above. A conventional tape-type configuration (Fig. S1) was applied in a preliminary experiment using PA-C gels (experiment 0). Here, the DGT gel was fixed on the Al sample by applying adhesive tape to all four sides of a polycarbonate membrane placed on top of the gel, while ensuring that the stack was free of air bubbles. A Petri dish filled with 40 ml NaCl was used for immersion of the tape-type configuration, whereas all other deployment parameters were identical to the piston-type deployment. The applied configurations, DGT gels, and deployment times for all experiments are provided in Table 1. All experiments were performed at 21 °C ± 1 °C.

### LA-ICP-MS analysis of DGT gels

Analyte mass loading distributions on dried DGT gels were analyzed using a nanosecond 213 nm solid-state Nd:YAG laser ablation system (NWR213, Elemental Scientific Lasers, USA) coupled to a ICP-MS/MS system (Agilent 8800, Agilent Technologies, JP), and a nanosecond 193 nm ArF excimer LA system (NWR193, ESI New Wave Research, USA) coupled to a quadrupole ICP-MS (NexION 2000B, PerkinElmer, USA). The performance of both systems was optimized daily for maximum signal-to-noise using gel standards. LA-ICP-MS analysis of DGT gels was performed in line scan mode with a total measurement time between 1.5 and 6 h per sample. Helium was used as carrier gas at flow rates of 0.8–0.9 L min^−1^ for aerosol transport and mixed with Ar gas prior to introduction into the ICP-MS. Signal intensities of ^13^C, ^27^Al, ^63^Cu, and ^66^Zn were recorded as counts in all analytical runs. Before sample analysis, at least three lines were ablated on each DGT blank and standard with line lengths ranging from 3 to 5 mm. Before the start and after the end of each line scan, a gas blank was recorded for 20–30 s to monitor the analyte background and washout signal [30]. A detailed compilation of the applied LA-ICP-MS parameters for all analyses is provided in the Supplementary Information (Table S1).

### Application of complementary imaging methods

Optical microscopy (StereoDiscovery V12/V20 and AxioImager M1, both Carl Zeiss Microimaging GmbH, DE) was used to visualize regions of localized corrosion on the Al samples after immersion, providing a reference for the selection of regions of interest (ROIs) on DGT gels for LA-ICP-MS analysis. The microstructure and chemical composition of the Al surface before and after immersion in NaCl and gel deployment in experiment 4 was assessed using a field emission scanning electron microscope (SEM; JEOL 7200 F; JEOL Germany GmbH, DE) equipped with a 100 mm^2^ silicon drift detector (Ultim Max 100; Oxford Instruments NanoAnalysis, DE) for energy-dispersive X-ray spectroscopy (EDX). The set electron acceleration voltage of 15 kV resulted in an EDX analysis depth of approximately 0.5 µm.

### Data evaluation and image generation

Data evaluation was conducted in Microsoft Excel. Outlier correction was applied to gas blank data points of ablated gel samples and ablation signal data points of gel blanks. This correction aimed to mitigate the impact of signal spikes on subsequent data analysis and enhance signal consistency. Outliers, defined as data points distant from the median, were identified using quartiles (*Q*_1_, *Q*_3_), interquartile range (IQR), and maximum (*Q*_3_ + 1.5 × IQR) and minimum (*Q*_1_ − 1.5 × IQR) values. Data points outside this range were excluded as outliers. The average of outlier-corrected gas blank signal intensities was then calculated and applied for gas blank correction. The resulting gas blank-corrected data points were normalized using ^13^C as internal standard [28, 33]. Each normalized data point was then converted to a DGT gel mass loading (*Γ*_DGT_) using the calibration function of a second-order polynomial regression for Al in experiment 4, or linear regression for Al, Cu, and Zn in experiments 1, 2, 3, and 4 (Cu and Zn only). *Γ*_DGT_-values were calculated according to Eq. 4:

4 $${\mathit\Gamma }_{{\text{DGT}}}= \frac{{m}_{a}}{A}$$

where *m*_a_ (ng) is the analyte mass and *A* (cm^2^) is the gel surface area. Outlier- and gas blank-corrected values of the gel blanks were used to calculate the method detection limit (MDL = 3 × standard deviation (*s*) of analyte signal on blank DGT gel) and method quantification limit (MQL = 10 × *s* of analyte signal on blank DGT gel). Fiji ImageJ (National Institute of Health, Bethesda, USA) [42] was used to generate pseudo-color images of ${\mathit\Gamma }_{{\text{DGT}}}$ with each pixel representing one data point, and InDesign (CS6, Adobe, San Jose, CA, USA) was used for final image arrangement as described elsewhere [30]. Fiji’s plugin Coloc 2 was used for Pearson’s correlation analysis (without thresholds).

## Results and discussion

### Performance of DGT gels for Al mapping

#### Mechanical properties

The mechanical properties of DGT gels controlled their handling and successful application. While polyacrylamide (PA) has been traditionally the most common matrix material in DGT gels, polyurethane (PU) exhibited superior mechanical properties for gel preparation, deployment, and subsequent LA-ICP-MS analysis. Consistent with previous studies [28, 29], the prepared PU gels had a highly homogeneous distribution of binding phases, facilitating their even deployment on the sample surface. Although being only ~ 100 µm thick, they remained tear-proof during handling for deployment, drying, and LA-ICP-MS analysis. In contrast, PA gels exhibited uneven settling of binding phases on one side of the gel, leading to rolling and complicating their even deployment on the sample surface. Despite their greater thickness (~ 400 µm), PA gels were more fragile during handling and more brittle after drying than PU gels, resulting in potential cracking of the gel surface upon the first laser impact in the LA system’s sample chamber, increasing spatial uncertainty in the analysis. Moreover, acrylamide, a basic component in the fabrication of PA gels, is classified as a group 2A carcinogen by the International Agency for Research on Cancer (IARC) [43], which should be replaced with non-toxic alternatives such as PU according to green chemistry principles [44, 45]. Consequently, PU gels emerged as the most favorable choice for DGT-based solute mapping in this study and hold promise for supporting future green DGT applications as well.

#### Shrinkage effects

Knowledge on shrinkage effects of DGT binding gels is essential for ensuring the accuracy of DGT-based solute mapping. Shrinkage leads to variability in the dimensions of the gel, potentially causing spatial distortion and altered diffusion dynamics, affecting the representation of the distribution of in situ solubilization features, especially in high-resolution applications. Therefore, potential shrinkage of the applied gels was assessed after immersion in NaCl for 24 h and subsequent gel drying.

**Fig. 2:**
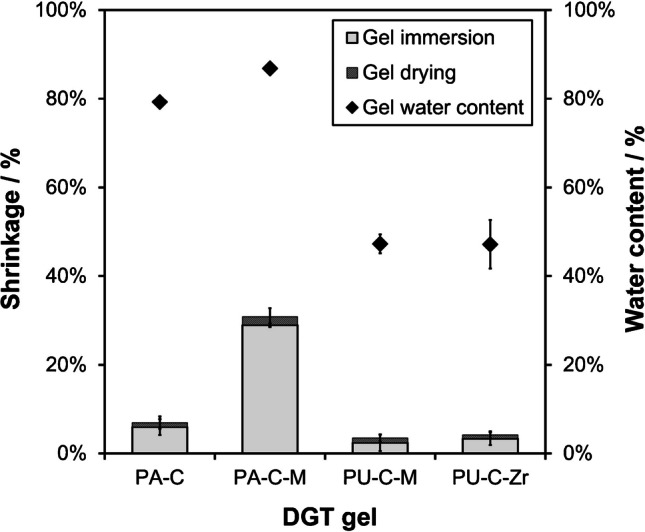
Average shrinkage (stacked bars) and water content (diamonds) of PA-C, PA-C-M, PU-C-M, and PU-C-Zr gels. Shrinkage is shown after gel deployment in NaCl solution (*w* = 1.5%, pH = 4.5) for 24 h (gray fill) and after subsequent gel drying (pattern fill) relative to the control group deployed in laboratory water type 1 in %. Error bars show *u*_c_ (*k* = 1) for shrinkage values (including the *s* of the sample (*n* = 2) and control (*n* = 2) group replicates) and *s* (*n* = 3) for water content values

Figure 2 shows that all gel types exhibited shrinkage upon immersion in NaCl as compared to control gels immersed in laboratory water type 1, whereas shrinkage during vacuum-assisted drying was insignificant with respect to the combined uncertainty (*u*_c_). However, shrinkage of PA gels was substantially higher than that of PU gels. This observation can be explained by osmotic effects, as PA gels exhibit higher gravimetric water contents than PU gels (Fig. 2) and hence release more water during immersion in NaCl. The most pronounced shrinkage effect was observed for PA-C-M gels, indicating a combined hydrophilic effect of both Metsorb and PA, resulting in the highest water content and strongest shrinkage amongst the tested DGT gel types. In comparison, PU-based hydrogels exhibit lower hydrophilicity [46], leading to a lower water content and consequently less shrinkage. Interestingly, previous work using PA-C-M gels for assessing dissolved Al concentrations in saline waters did not report shrinkage [47, 48]. In our study, the introduction of Metsorb with a 5-µm particle size may have enhanced water uptake and related osmotic effects as compared to the 50-µm particle size Metsorb used in the previous work, contributing to more noticeable shrinkage compared to the original gel formulation. This observation underscores the importance of pre-conditioning DGT gels as an essential procedural step to prevent spatial distortion in DGT-based solute mapping.

#### Binding characteristics

Uptake (*f*_u_) and elution efficiencies (*f*_e_) of Al by PA-C-M and PU-C-M gels were evaluated in a mass balance experiment using NaCl solutions (*w* = 1.5%, pH = 4.5) containing a total Al mass of either 12.1 µg ± 0.1 µg (solution 1) or 49.6 µg ± 0.1 µg (solution 2). Results are compiled in Table S2, showing a similar performance of both gel types under the experimental conditions. While Al uptake was high for solution 1 (*f*_u_ = 0.84–0.87), a substantial decrease was observed for solution 2 (*f*_u_ = 0.38–0.46), indicating limitations of these gels for quantitative sampling of Al beyond a capacity estimate of 10 µg per gel disc, equating to *Γ*_DGT_(Al) = 2.2 µg cm^−2^. Conversely, the Al elution efficiency increased from gels deployed in solution 1 (*f*_e_ = 0.70–0.77) to gels deployed in solution 2 (*f*_e_ = 0.84–0.93), suggesting that Al elution is dependent on the Al mass loading on the gel. Although these results show that both PA-C-M and PU-C-M are generally applicable for sampling Al under the experimental conditions, the relatively low binding capacity and variable elution efficiency introduce additional uncertainty when using these gels for DGT-based Al solute mapping.

**Fig. 3:**
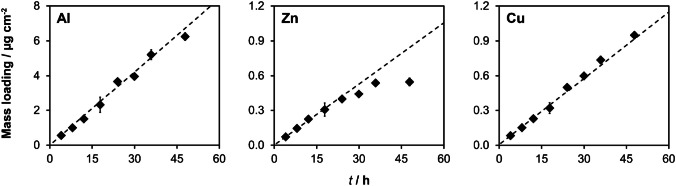
Uptake of Al, Zn, and Cu by PU-C-Zr gels in standardized DGT bulk sampling experiment. DGT samplers containing PU-C-Zr gels were immersed in NaCl solution (*w* = 1.5%, pH = 4.5) for varying times (*t* = 4–48 h). Dashed lines show the theoretical accumulation under perfect-sink conditions. Diamonds show experimental values. Error bars are *s* (*n* = 3) or smaller than the symbol size if not visible

The third mixed binding gel, PU-C-Zr, was previously characterized for high-resolution sampling of multiple anions and cations including Zn and Cu [28]. However, until now, this gel has not been characterized for quantitative sampling of dissolved Al. Therefore, the time-dependent uptake of Al, Zn, and Cu by PU-C-Zr gels was assessed in a standardized DGT bulk sampling experiment. Gel digestion was applied to fully recover the bound analyte fractions [28], hence eliminating uncertainties associated with gel elution and non-quantitative recovery. Figure 3 shows that the PU-C-Zr DGT accumulated Al and Cu linearly for all deployment times, confirming perfect-sink conditions. The measured Al and Cu mass loadings were in excellent agreement with those predicted from DGT theory as shown by an average *Γ*_measured_/*Γ*_predicted_ ratio of 0.97 ± 0.07 for Al and 1.01 ± 0.06 for Cu. Noteworthy, the measured maximum Al mass loading (*Γ*_DGT_ = 6.25 µg cm^−2^) was higher than that previously reported for different gels [35], suggesting a higher Al binding capacity. For Zn, the measured mass loadings were also in agreement with the predicted values for deployment times between 4 and 36 h (*Γ*_measured_/*Γ*_predicted_ = 0.95 ± 0.08). However, after 48 h, a substantial underestimation of Zn by PU-C-Zr DGT was observed (*Γ*_measured_/*Γ*_predicted_ = 0.65), indicating competitive desorption by Al and Cu and hence a Zn capacity estimate of *Γ*_DGT_ = 0.54 µg cm^−2^ under the experimental conditions.

### Effect of DGT deployment conditions on Al mapping

#### Deployment configuration

**Fig. 4:**
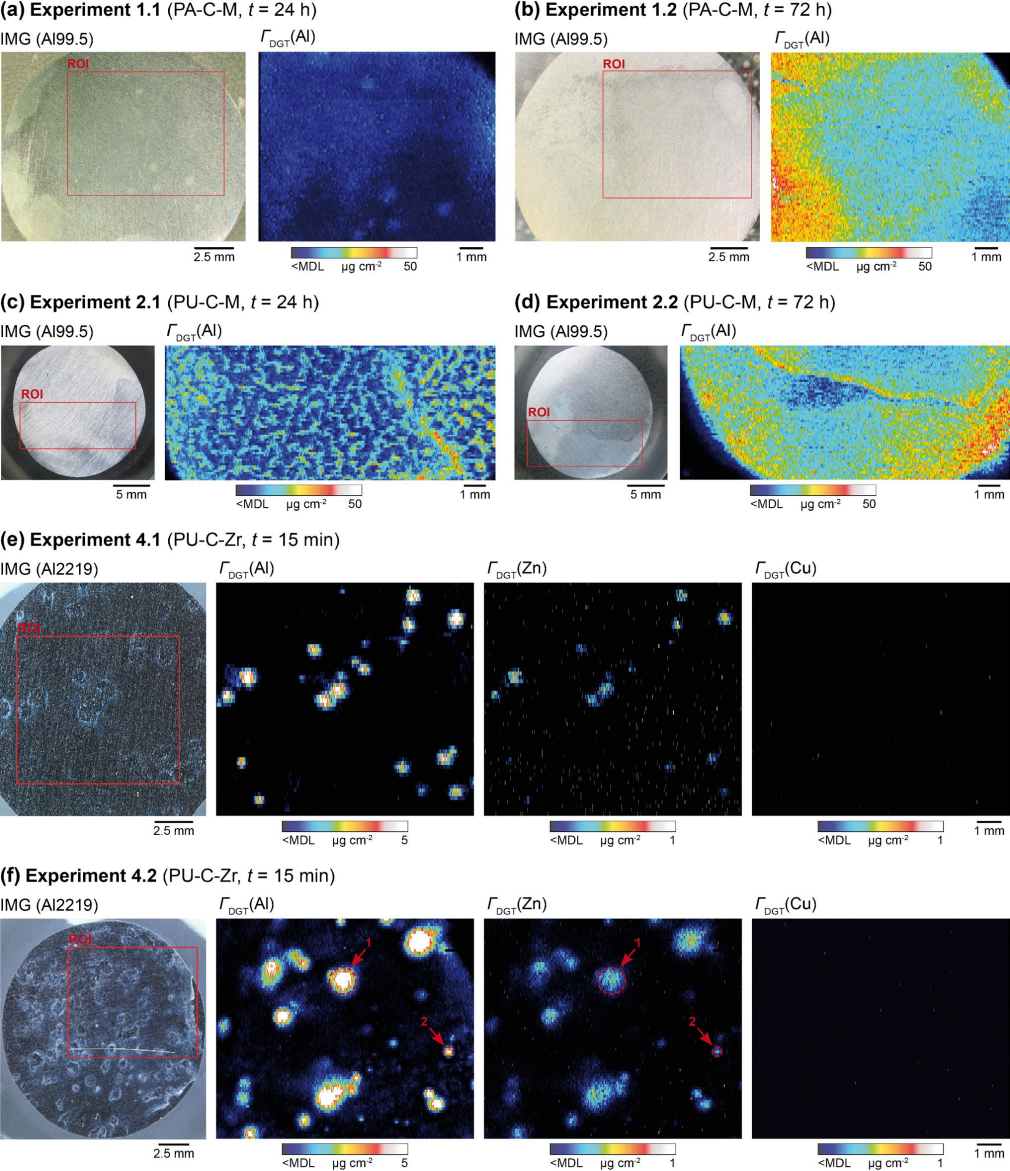
Solute maps showing elemental solubilization and release from Al materials exposed to NaCl solution (*w* = 1.5%, pH = 4.5) after 24 h (**a**) and 72 h (**b**) of PA-C-M deployment on Al99.5 in experiment 1, 24 h (**c**) and 72 h (**d**) of PU-C-M deployment on Al99.5 in experiment 2, and 15 min of PU-C-Zr deployment on Al2219 in replicates 1 (**e**) and 2 (**f**) of experiment 4. The black to white color scale represents a sequential increase in *Γ*_DGT_ (µg cm.^−2^). Red frames in photographs (IMGs) indicate the analyzed ROIs on Al samples. Red arrows and dashed circles in *Γ*_DGT_(Al) and *Γ*_DGT_(Zn) maps in (f) indicate ROIs 1 and 2 shown in Fig. 7a and b, respectively. Results of experiments 0 and 3 are shown in the Supplementary Information (Figs. S2 and S3)

A new piston-type deployment configuration (Fig. 1) was developed aiming at DGT gel application on metal samples with high positional reproducibility during in situ solute sampling. The configuration uses the conventional DGT piston cap to fix the gel on a metal surface embedded in epoxy. Thereby, the use of adhesive tape is avoided, which may damage the gel, introduce elemental contamination, or, importantly for Al corrosion, lead to the formation of an occluded interstice in which the aqueous medium is trapped and not renewed, causing crevice corrosion [9, 49]. The latter effect was also observed in the preliminary experiment of this study (experiment 0), where the conventional tape-type deployment configuration for gel fixation on the sample surface was used (Fig. S1). Here, the crevice between the metal and the tape led to stagnant conditions, preventing renewal of the NaCl solution. This led to clear patterns of millimeter-scale crevice corrosion on the sample surface, which were also reflected in the corresponding Al solute maps (Fig. S2). Using the piston-type deployment configuration, no direct contact between the DGT cap and Al sample edges was established, which effectively prevented crevice formation and resulted in a different, more localized Al solute distribution, as shown in the Al solute maps of experiments 1–4 (Figs. 4 and S3). Thus, the deployment configuration of the DGT gel was found to be a primary parameter affecting the corrosion reactions occurring at the Al sample surface. Although previous modelling and experimental work showed that the DGT gel cannot directly induce localized maxima or minima of solute release [50, 51], deployment of the gel on the material surface modulates the local solution flow affecting the exchange of reaction products. This needs to be considered when investigating aqueous corrosion processes that are directly dependent on the rates of interaction between the material surface and the surrounding solution.

#### Sample surface roughness

To achieve solute sampling by DGT at high spatial resolution, it is beneficial to use a sample with minimum surface roughness to ensure a direct contact between the DGT gel and the sample and hence immediate binding of solubilized metal fractions without lateral diffusion during uptake. Therefore, two different metallographic approaches of surface polishing after grinding were compared to identify potential differences in the resulting surface roughness of the investigated Al materials.

**Fig. 5:**
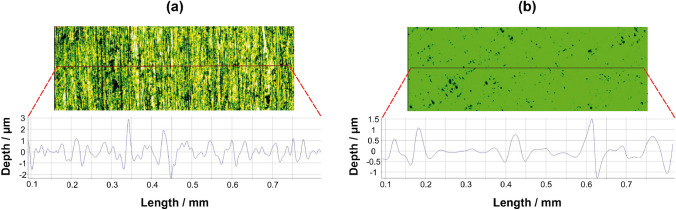
Surface roughness measurements for 1-µm diamond-polished Al99.5 (**a**) and for colloidal silica-polished Al2219 (**b**)

The results (Fig. 5) showed that, depending on the polishing approach, the resulting surface roughness can be significantly different. Using the first approach (1-µm diamond-polished), parallel scratches with a surface roughness ranging in depth between − 2.0 and 3.0 µm (*∆*_depth_ = 5 µm) were obtained (Fig. 5a). In contrast, the second approach (colloidal silica-polished) resulted in a substantially lower surface roughness with values ranging between 1.5 µm and − 1.0 µm (*∆*_depth_ = 2.5 µm; Fig. 5b). It should be noted that despite using two different Al materials in this investigation (Al99.5 and Al2219), the resulting polishing effect is expected to be identical for both given their overall similar polishing properties [52]. The influence of the residual surface roughness is possibly also exemplified in the Al solute map obtained after 24 h of PU-C-M deployment on Al99.5 in experiment 2 (Fig. 4c). Here, a highly heterogeneous pattern of Al was observed as compared to experiment 1, where the identical deployment conditions but a PA-C-M gel were applied (Fig. 4a). This can be explained by the fact that PU gels are thinner and exhibit a lower water content than PA gels (Fig. 2), resulting in a higher susceptibility to sampling artifacts due to non-perfect gel-sample contact and hence potential formation of small-scale voids during deployment. Given the overall benefits of using PU as a gel matrix as compared to PA (see discussion in “Performance of DGT gels for Al mapping”), future work should investigate the manufacturing possibilities and binding characteristics of thicker PU gels to facilitate their even contact with a sample surface with residual surface roughness, which often cannot be avoided in practice, especially when aiming at field applications on metallic structures.

#### Deployment time

Four different DGT deployment times, including 15 min, 80 min, 24 h, and 72 h, which corresponded to the time of material immersion in NaCl solution, were applied to investigate its effect on the mapped Al distribution and mass loading on DGT gels. First, PA-C-M and PU-C-M gels were deployed both for either 24 h or 72 h on Al99.5 samples in experiments 1 and 2. The resulting Al solute maps are shown in Fig. 4a and b (experiment 1) and Fig. 4c and d (experiment 2). Clearly, the deployment time had a direct impact on Al mapping in these experiments, with generally lower *Γ*_DGT_(Al) values for 24 h as compared to 72 h of deployment. For example, after 24 h of PA-C-M deployment, the median *Γ*_DGT_(Al) over the full ROI was 5.57 µg cm^−2^, which increased to 18.1 µg cm^−2^ after 72 h (Fig. 4a and b). Interestingly, the median *Γ*_DGT_(Al) for 24 h of PU-C-M deployment was 94% higher than for the PA-C-M gel (Fig. 4a and c), whereas after 72 h, the difference in *Γ*_DGT_(Al) between the different gels only accounted for 3% (Fig. 4b and d). This suggests different reactivity of Al99.5 when using PA or PU gel types characterized by different hydrophilicity (Fig. 2). However, given that all values were above the estimated Al capacity limit of 2.2 µg cm^−2^ for both gel types, these findings need to be interpreted with caution, because uptake was likely non-quantitative.

Therefore, shorter deployment times were applied in the following experiments 3 and 4 aiming et al. uptake below the binding gel capacity. Moreover, the use of Al2219, which is characterized by higher susceptibility to pitting corrosion as compared to Al99.5 [14], was expected to result in higher corrosion rates in these experiments. The resulting Al solute maps are shown in Fig. S3 (experiment 3), as well as Fig. 4e and f (experiment 4). Although, a more heterogeneous Al distribution was observed after 80 min of PA-C-M deployment as compared to 24 h and 72 h of PA-C-M deployment, intensified corrosive dissolution of Al2219 still led to local saturation of the binding phases in the gel and thus non-quantitative Al uptake as evidenced by local *Γ*_DGT_(Al) maxima up to 36.5 µg cm^−2^ (Fig. S3). Consequently, the deployment time was further reduced to only 15 min for the deployment of PU-C-Zr gels. Here, distinct areas of increased *Γ*_DGT_(Al) were mapped in isolated zones of the gel, revealing steep gradients of Al solubilization from the Al2219 into the gel (Fig. 4e and f). In addition, maximum *Γ*_DGT_(Al) values were 4.94 µg cm^−2^ and 5.94 µg cm^−2^ in replicates 1 and 2 of experiment 4, respectively, and thus below the previously measured maximum *Γ*_DGT_(Al) of 6.25 µg cm^−2^ where Al uptake was still quantitative (Fig. 3).

Thus, as shown in previous work [33], careful selection of the deployment time depending on the specific material reactivity is crucial when designing experiments for DGT-based solute mapping in aqueous corrosion studies. Thereby, spatial resolution and solute quantification capabilities are controlled. If in doubt, preliminary experiments should be performed to assess optimum deployment times where analyte uptake is above the method’s limit of detection but below its binding capacity.

### Quantitative mapping of Al release at sub-picogram levels

As discussed in the previous sections, deployment of PU-C-Zr gels on Al2219 for 15 min (experiment 4) showed the best performance for quantitative mapping of aqueous Al corrosion at high spatial resolution. Therefore, the results of this experiment are further discussed in detail below.

#### Calibration

**Fig. 6:**
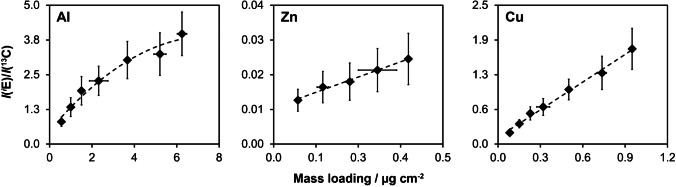
Exemplary LA-ICP-MS calibration for Al, Zn, and Cu using PU-C-Zr gel standards. Dashed lines show the fitted regression lines for Al (second-order polynomial) as well as Cu and Zn (linear). Error bars show *s* (*n* = 3) of the mass loading (*x*-axis) and *u*_c_ (*k* = 1) of the gas blank-corrected and ^13^C-normalized signal intensity (*y*-axis), including the within-line (*n* =  ~ 500 − 1000) and in-between-line (*n* = 3) repeatability. If not visible, error bars are smaller than the symbol size. *I*(^*i*^E) on the *y*-axis represents the gas blank-corrected signal intensity of ^27^Al, ^66^Zn, and ^63^Cu, respectively

An exemplary LA-ICP-MS calibration for Al, Zn, and Cu using PU-C-Zr gel standards is shown in Fig. 6. A second-order polynomial regression was used for *Γ*_DGT_(Al) values between 0.56 µg cm^−2^ and 6.25 µg cm^−2^, resulting in high correlation coefficients for all analyses (*R*^2^ = 0.96–0.99). Nonlinear behavior at high mass loadings was previously reported for P analysis using a ferrihydrite DGT LA-ICP-MS method [53]. The authors suggested a possible interaction between Fe oxide, which is the binding phase in ferrihydrite DGT gels, and P in the aerosol produced by the laser, resulting in decreasing signal intensities with increasing P mass loadings. Such an interaction could have also occurred in the present study, where Zr-hydroxide particles might have reacted with Al in the aerosol, leading to increased deposition and agglomeration of particles during transportation to the ICP-MS at high Al mass loadings. No further investigation of this effect was conducted because the presented results enabled precise calibration and were hence fit for purpose. For Cu and Zn, linear calibration curves were obtained for *Γ*_DGT_(Cu) between 0.08 µg cm^−2^ and 0.95 µg cm^−2^ (*R*^2^ = 0.99) and *Γ*_DGT_(Zn) between 0.07 µg cm^−2^ and 0.40 µg cm^−2^ (*R*^2^ = 0.99), which was well in line with previous work reporting DGT LA-ICP-MS calibrations for Cu and Zn using PU-C-Zr gels [28].

#### Detection limits

The MDLs and MQLs for Al, Zn, and Cu, expressed as mass loadings *Γ*_DGT_ obtained by analyzing blank PU-C-Zr gels, are compiled in Table S3. All values were in the low ng cm^−2^ range, with MDLs ranging between 0.41 ng cm^−2^ for Cu and 83.8 ng cm^−2^ for Zn, and MQLs ranging between 1.36 ng cm^−2^ for Cu and 279 ng cm^−2^ for Zn. MDLs and MQLs for Al were in between those for Zn and Cu, ranging between 2.51 ng cm^−2^ and 7.22 ng cm^−2^, and 8.36 ng cm^−2^ and 24.1 ng cm^−2^, respectively. Overall, these values were in line with those reported previously for PU-C-Zr and different gels [28, 35].

When converting the measured mass loadings to absolute analyte masses for an exemplary area of 100 µm × 100 µm (*A* = 0.01 mm^2^), MDLs and MQLs of ≤ 0.72 pg and ≤ 2.41 pg of Al, ≤ 8.38 pg and ≤ 27.9 pg of Zn, and ≤ 0.12 pg and ≤ 0.41 pg of Cu, respectively, were obtained. This demonstrates the unique capability of the developed method for quantitative mapping of localized elemental solubilization down to sub-picogram levels, which was previously not possible using existing surface-sensitive analysis techniques in corrosion research. For instance, when considering an Al alloy with a typical density of 2.7 g cm^−3^ and uniform Al dissolution across an area of 1 mm^2^, the removal of an Al surface layer down to 1-nm thickness can still be detected. Auspiciously, precleaning of the Chelex and Zr(OH)_4_ binding phases prior to impregnation into the gel could be applied to further lower the method’s detection capability, potentially enabling measurements of single femto- to attograms of elemental solute release.

#### Patterns of elemental solubilization

The calibrated solute maps obtained after deployment of PU-C-Zr gels for 15 min on Al2219 in two replicates (Fig. 4e and f) revealed highly heterogeneous patterns of localized *Γ*_DGT_(Al) and *Γ*_DGT_(Zn) increases, forming multiple distinct solubilization hotspots across the full ROI. Pearson’s correlation coefficients between *Γ*_DGT_(Al) and *Γ*_DGT_(Zn) were 0.56 and 0.84 for experiments 4.1 and 4.2, respectively, indicating their systematic co-localization. For Cu, no areas of increased *Γ*_DGT_(Cu) were observed in any of the experimental replicates, as shown by *Γ*_DGT_(Cu) values consistently below the MDL.

The *Γ*_DGT_(Al) and *Γ*_DGT_(Zn) increases were highly localized as shown by their confined spatial expansion with approximate diameters between 50 and 1000 µm. In their immediate surroundings, a white precipitate was observed in the photographs of the material surface (outer left in Fig. 4e and f), indicating the formation of Al(OH)_3_, a typical corrosion product resulting from hydrolysis of Al chlorides [10, 14]. Noteworthy, the areas of increased *Γ*_DGT_(Al) and *Γ*_DGT_(Zn) showed a more confined spatial expansion, and the material surface showed a less dense formation of Al(OH)_3_ in experiment 4.1 as compared to experiment 4.2. This suggests intensified corrosion in the latter experiment despite identical deployment conditions. A possible explanation for this observation is a higher corrosive reactivity of the Al2219 sample used in experiment 4.2, reflecting a typical scatter of corrosion processes. Moreover, the storage time of the metallographic sections used in experiments 4.1 (83 days) and 4.2 (153 days) was different, which might have influenced their passive state.

**Fig. 7:**
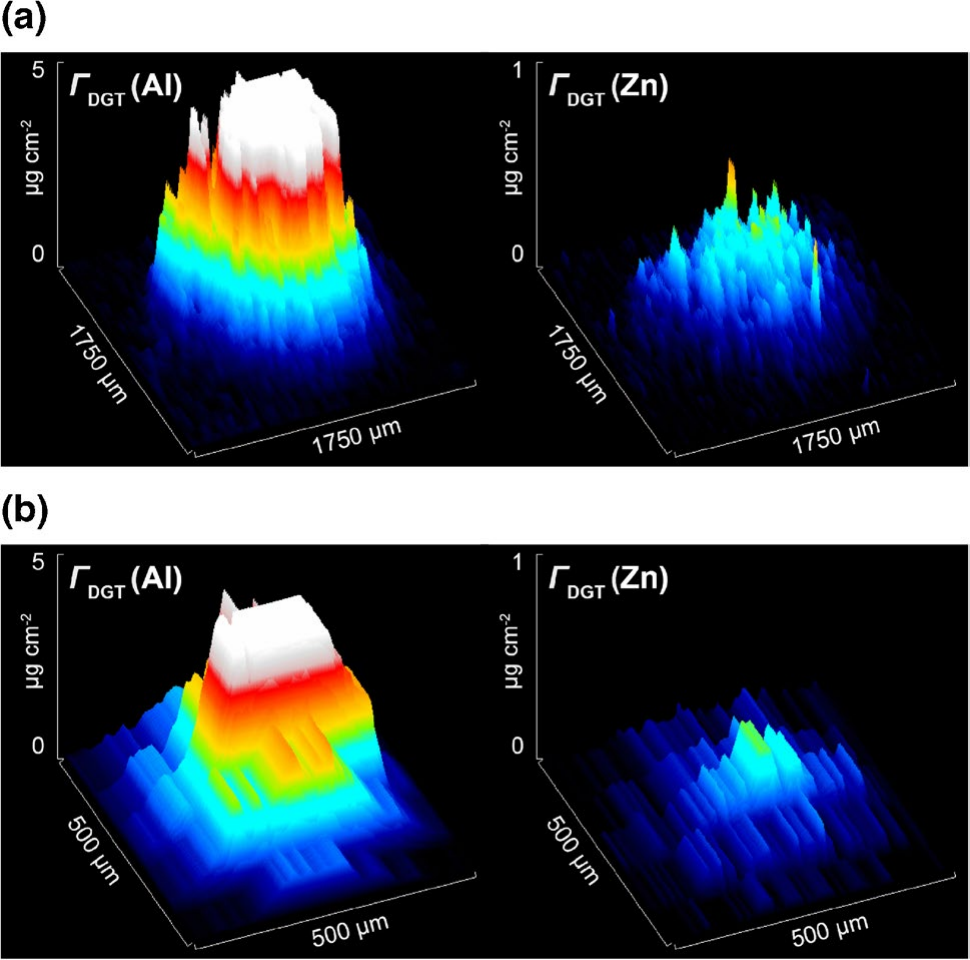
Solute plots of *Γ*_DGT_(Al) and *Γ*_DGT_(Zn) including ROIs 1 (**a**) and 2 (**b**) in experiment 4.2 depicting the localized release of analytes. Locations of the ROIs are indicated with red arrows and dashed circles in the Al and Zn solute maps in Fig. 4f. The black to white color scale represents a sequential increase in *Γ*_DGT_ (µg cm^−2^)

The observed pattern shows that the origin of the mapped Al and Zn co-solubilization was pitting corrosion. Given that the mapped areas of increased *Γ*_DGT_(Al) and *Γ*_DGT_(Zn) were mostly larger than those expected for pit clusters with typical surface opening diameters < 100 µm [17], it is likely that they are a result of propagating sub-surface solute release, leading partly to local saturation of binding sites at the microscale and subsequent lateral within-gel diffusion. In addition, the spatial resolution of the obtained solute maps is limited by the sensitivity of the applied quadrupole ICP-MS instrumentation, requiring a minimum laser spot size of 50 µm for adequate signal-to-noise separation. Yet, as exemplarily shown for two selected ROIs in experiment 4.2 (Fig. 7a and b), the absolute mass of solubilized Al and Zn after 15 min of immersion, calculated via multiplication of the median *Γ*_DGT_ times the ROI area (*A*_ROI 1_ = 0.98 mm^2^; *A*_ROI 2_ = 0.06 mm^2^), could be accurately quantified at 27.1 ng of Al and 1.68 ng of Zn for ROI 1, and 2.01 ng of Al and 0.10 ng of Zn for ROI 2. This shows that the new approach effectively enables in situ, time-integrated, and high-resolution mapping of multiple elemental solutes and diffusion patterns formed during aqueous corrosion. Simultaneous application of shorter deployment times (e.g., 1–5 min) and application of ICP-MS instrumentation with enhanced sensitivity (e.g., sector-field ICP-MS) may enable future work to assess multielement dissolution dynamics of single pitting events occurring at < 50 µm.

#### Application of complementary imaging methods

**Fig. 8:**
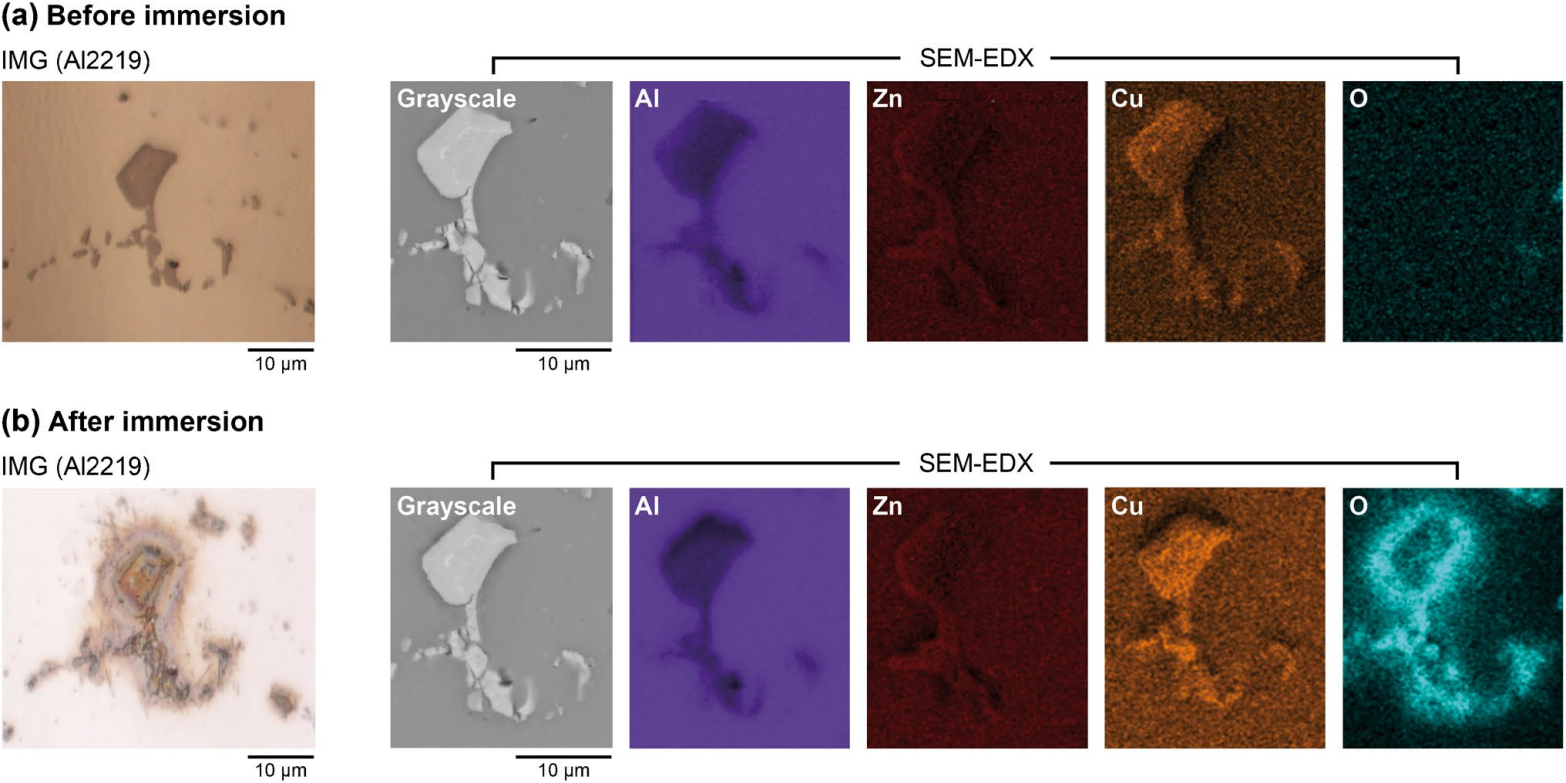
Images of a soluble Al_2_Cu particle in the Al2219 matrix obtained by optical microscopy (outer left) and SEM–EDX before (**a**) and after (**b**) immersion in NaCl solution and DGT deployment for 15 min in experiment 4.2. Color scales in SEM–EDX images correspond to relative elemental contents from low (dark) to high (bright)

In addition to the high-sensitivity mapping of localized analyte release by DGT LA-ICP-MS, optical microscopy and SEM–EDX were applied on a selected ROI on the Al2219 sample used in experiment 4.2 before and after its immersion in NaCl and PU-C-Zr gel deployment for 15 min. The ROI was selected to include the interface between the Al matrix and an intermetallic particle, which was identified as a coarse soluble Al_2_Cu particle based on its elemental composition measured at an analysis depth of approximately 0.5 µm by SEM–EDX (Fig. 8a), as well as literature data on Al2219 [38]. After immersion and gel deployment, visible changes in the interfacial microstructure and elemental composition were observed (Fig. 8b), clearly indicating localized aqueous corrosion. As shown by elevated levels of Al, Cu, and especially O at the particle surface and in its immediate surroundings, insoluble Al(OH)_3_ and copper(I) chloride (CuCl) were formed during corrosion. This is consistent with previous work [14, 54] showing that Al-Cu particles act as cathode with respect to the Al matrix, causing intensified pitting attack at the interface region by galvanic and/or chemical interactions. Cathodic passivation of the Al_2_Cu particle surface also explains the absence of Cu solubilization, whereas anodic oxidation of Al and Zn in the alloy matrix promoted their effective release into solution as shown in solute maps (Fig. 4e and f).

## Conclusion

This is the first time that the combination of DGT with LA-ICP-MS has been used to simultaneously localize and quantify the solubilization and diffusion of multiple elements during aqueous corrosion of Al alloys. The observation of intensified Al and Zn release with a concomitant absence of Cu solubilization confirmed that pitting corrosion occurs at intermetallic interfaces localized by complimentary SEM–EDX. Thus, the limitation of existing methods only capable of visualizing surface alterations without detecting localized elemental dissolution processes has been successfully overcome. The approach has been comprehensively evaluated in laboratory experiments, demonstrating its unique capability to assess the in situ distribution of corrosive elemental solute release at high (50 µm) spatial resolution and with unprecedented detection limits down to sub-picogram levels when using PU-C-Zr gels. The main advantages of this gel type are its tear-proof handling, low susceptibility to shrinkage, compliance with green chemistry, and quantitative binding characteristics with a higher Al capacity as reported for different gels. By the application of the newly developed piston-type deployment configuration, standardization of DGT-based solute measurements for corrosion research becomes possible, providing a basis for data reproducibility and comparability with defined sampling parameters. This will support potential applications focusing on the investigation of localized corrosion dynamics in aqueous environments by facilitating time series deployments of DGT gels on the same sample specimen. Thereby, the local and temporal effects of solute partitioning and surface passivation on aqueous corrosion can be better understood, potentially guiding the design of future multi-component Al alloys towards enhanced material longevity and technological sustainability. In addition to its relevance for material characterization, the capability of the new approach for selective and spatially resolved measurements of labile Al solute fractions has also great potential for environmental applications, where PU-C-Zr gels in combination with LA-ICP-MS can now be used to investigate small-scale Al mobilization processes in natural waters, sediments, and soils. Thereby, the approach benefits researchers and engineers from a wide range of fields who are currently limited to ex situ techniques for assessing material characteristics and environmental fluxes of Al at high spatial resolution.

### Supplementary Information

Below is the link to the electronic supplementary material.

Supplementary file1 (PDF 580 KB)

## Data Availability

The datasets generated during the current study are available from the corresponding author on reasonable request.
