# Supplementary material for: Mapping elemental solutes at sub-picogram levels during aqueous corrosion of Al alloys using diffusive gradients in thin films (DGT) with LA-ICP-MS
Source: Anal Bioanal Chem. 2024 Apr 16;416(14):3373–88. doi: 10.1007/s00216-024-05288-8 (PMC11106204; doi:10.1007/s00216-024-05288-8)
Supplement: Supplementary file 1 — Supplementary file1 (PDF 580 KB) [file 216_2024_5288_MOESM1_ESM.pdf]

Supplementary Information

Analytical and Bioanalytical Chemistry

Mapping elemental solutes at sub-picogram levels during aqueous corrosion of Al alloys using diffusive gradients in thin films (DGT) with LA-ICP-MS

Gulnaz Mukhametzianova[1,2,§], Stefan Wagner[1,2,§,*], Magdalena Eskinja[1], Masoud Moshtaghi[1,3], Gregor Mori[1], Thomas Prohaska[1]

[1]Montanuniversität Leoben, Department of General, Analytical and Physical Chemistry, Chair of General and Analytical Chemistry, Franz-Josef-Strasse 18, 8700 Leoben, Austria

[2]Montanuniversität Leoben, Christian Doppler Laboratory for Inclusion Metallurgy in Advanced Steelmaking, Franz-Josef-Strasse 18, 8700 Leoben, Austria

[3]Laboratory of Steel Structures, LUT University, P.O. Box 20, Lappeenranta 53851, Finland

[§]These authors contributed equally to this work.

*Corresponding author: stefan.wagner@unileoben.ac.at

Table of contents

1. Materials and methods S-2

1.1 Figure S1: Tape-type deployment configuration S-2

1.2 Table S1: LA-ICP-MS parameters S-3

2. Results and discussion S-4

2.1 Table S2: Al uptake and elution by PA-C-M and PU-C-M gels S-4

2.2 Figure S2: Solute maps of Al in experiment 0 ..... S-5

2.3 Figure S3: Solute map of Al in experiment 3 ..... S-6

2.4 Table S3: DGT LA-ICP-MS detection limits using PU-C-Zr gels S-7

1. Materials and methods

1.1 Figure S1: Tape-type deployment configuration

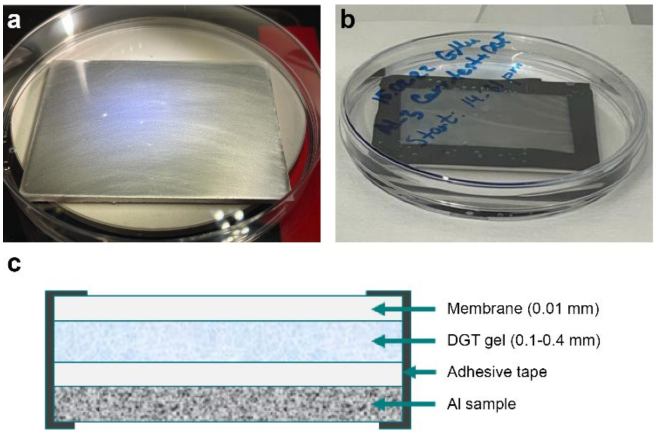

Fig. S1. Tape-type deployment configuration used in the preliminary experiment 0, including Al sample before (a) and during (b) immersion in NaCl solution and gel deployment, as well as cross-section through the deployment assembly showing the different components and their approximate thicknesses (c; not to scale).

1.2 Table S1: LA-ICP-MS parameters

Table S1. Operation parameters and instrumentation used for LA-ICP-MS analysis of DGT gels in all experiments

**Experiment**	**0**		**1**		**2**		**3**	**4**	
Sub-experiment	**0.1**	**0.2**	**1.1**	**1.2**	**2.1**	**2.2**	**-**	**4.1**	**4.2**
DGT gel	**PA-C**		**PA-C-M**		**PU-C-M**		**PA-C-M**	**PU-C-Zr**	
Deployment time	**24 h**	**72 h**	**24 h**	**80 min**	**24 h**	**72 h**	**80 min**	**15 min**	**15 min**
**Laser parameters**									
Laser	NWR213 (ESI)		NWR193 (ESI)		NWR193 (ESI)		NWR213 (ESI)	NWR213 (ESI)	
Aperture	XYR	XYR	IVA	IVA	IVA	IVA	XYR	IVA	XYR
Spot size / µm	60	60	75	75	75	75	60	50	50
Scan speed / µm s^{-1}	60	60	75	75	75	75	60	50	50
Repetition rate / Hz	20	20	20	20	20	20	20	20	20
Energy / %	45	45	30	30	15	15	20	20	20
Line scan distance / µm	12000	8000	10000	10000	15000	15000	5000	10000	10000
Interline distance / µm	100	100	100	100	100	100	150	150	100
Carrier gas (He) / l min^{-1}	0.8	0.8	0.9	0.9	0.9	0.9	0.9	0.9	0.9
**ICP-MS parameters**									
ICP-MS	8800 (Agilent)		NexION 2000 (Perkin Elmer)		NexION 2000 (Perkin Elmer)		8800 (Agilent)	8800 (Agilent)	
Measured isotopes	^{13}C, ^{24}Mg, ^{26}Mg, ^{27}Al, ^{63}Cu, ^{64}Zn, ^{65}Cu, ^{66}Zn	^{13}C, ^{26}Mg, ^{27}Al, ^{52}Cr, ^{54}Cr, ^{55}Mn, ^{57}Fe, ^{63}Cu, ^{66}Zn	^{13}C, ^{26}Mg, ^{27}Al, ^{48}Ti, ^{54}Cr, ^{55}Mn, ^{57}Fe, ^{60}Ni, ^{63}Cu, ^{66}Zn, ^{69}Ga, ^{88}Sr, ^{89}Y, ^{180}Hf, ^{208}Pb		^{13}C, ^{26}Mg, ^{27}Al, ^{48}Ti, ^{54}Cr, ^{55}Mn, ^{57}Fe, ^{60}Ni, ^{63}Cu, ^{66}Zn, ^{69}Ga, ^{88}Sr, ^{89}Y, ^{178}Hf, ^{208}Pb		^{13}C, ^{24}Mg, ^{26}Mg, ^{27}Al, ^{51}V, ^{55}Mn, ^{57}Fe, ^{63}Cu, ^{66}Zn	^{13}C, ^{27}Al, ^{56}Fe, ^{63}Cu, ^{65}Cu, ^{66}Zn	
Integration time per *m/z* / s	0.005-0.02		0.005-0.05		0.005-0.05		0.005-0.02	0.005-0.025	
Total acquisition time / s	0.1548	0.1929	0.4530		0.4530		0.1202	0.1497	
Length per datapoint / µm	9.288	11.574	33.975		33.975		7.212	7.485	
Sampler/skimmer cone	Ni		Ni		Ni		Ni	Ni	
RF power / W	1550		1600		1600		1550	1550	
Plasma gas (Ar) / l min^{-1}	15		18		18		15	15	
Measurement time / h	3.8	4.8	3.6	3.9	3.9	3.8	2.1	4.3	5.7
Analyzed area / mm^2	62	71	79	85	92	89	45	89	85

2. Results and discussion

2.1 Table S2: Al uptake and elution by PA-C-M and PU-C-M gels

Table S2. Mass uptake and elution as well as uptake (f_u) and elution (f_e) efficiencies of PA-C-M and PU-C-M gels for Al [a]

Al mass in immersion solution	PA-C-M				PU-C-M			
	Mass uptake	f_u	Mass elution	f_e	Mass uptake	f_u	Mass elution	f_e
µg	µg		µg		µg		µg	
12.1 ± 0.1	10.5 ± 0.2	0.87 ± 0.02	7.36 ± 0.13	0.70 ± 0.02	10.3 ± 0.6	0.84 ± 0.05	7.89 ± 0.36	0.77 ± 0.01
49.6 ± 0.1	23.0 ± 1.1	0.46 ± 0.02	19.2 ± 0.6	0.84 ± 0.03	18.6 ± 2.4	0.38 ± 0.05	17.4 ± 2.1	0.93 ± 0.01

[a] Errors are *s* of the replicates (n = 3)

2.2 Figure S2: Solute maps of Al in experiment 0

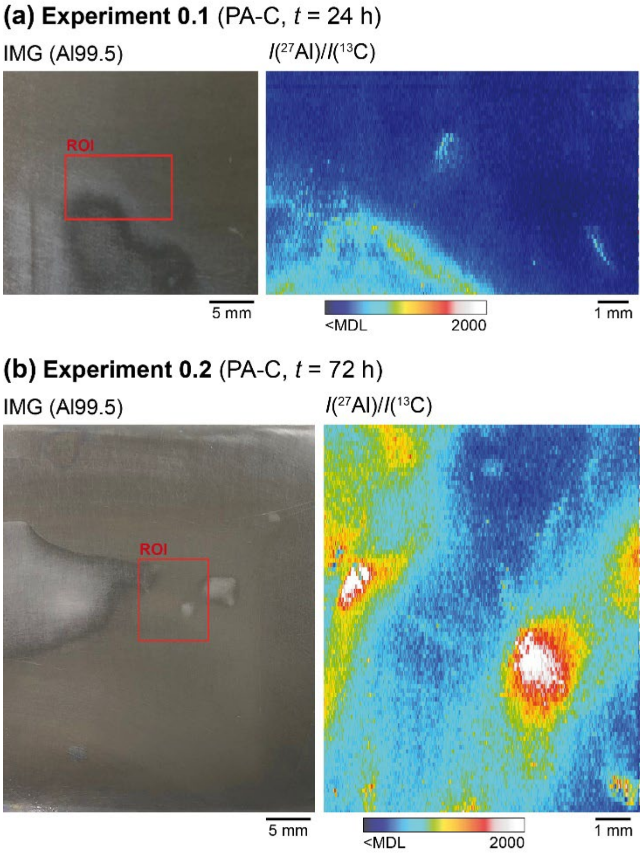

Fig. S2. Solute maps showing Al solubilization and release from Al99.5 exposed to NaCl solution (w = 1.5 %, pH = 4.5) after 24 h (a) and 72 h (b) of PA-C deployment in the preliminary experiment 0. The black to white color scale represents a sequential increase in the $^{27}Al/^{13}C$-normalized intensity (I) ratio. Red frames in the photographs (IMGs) indicate the analyzed regions of interest (ROIs) on Al99.5 samples. The presence of crevice corrosion is visible in the IMGs in areas with different color shading as compared to the bulk material.

2.3 Figure S3: Solute map of Al in experiment 3

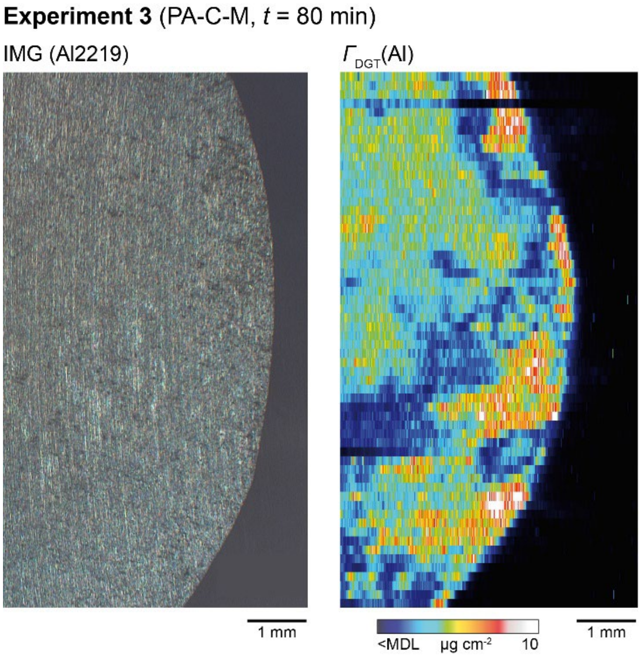

Fig. S3. Solute maps showing Al solubilization and release from Al2219 exposed to NaCl solution (w = 1.5 %, pH = 4.5) after 80 min of PA-C-M deployment in experiment 3. The black to white color scale represents a sequential increase in Γ_{DGT} (µg cm^{-2}). The photograph (IMG) covers the complete analyzed ROI on the Al2219 sample.

2.4 Table S3: DGT LA-ICP-MS detection limits using PU-C-Zr gels

Table S3. Method detection limits (MDLs) and method quantification limits (MQLs) for Al, Zn, and Cu measurements by DGT LA-ICP-MS using PU-C-Zr gels

Experiment	Al		Zn		Cu	
	MDL	MQL	MDL	MQL	MDL	MQL
	ng cm^{-2}	ng cm^{-2}	ng cm^{-2}	ng cm^{-2}	ng cm^{-2}	ng cm^{-2}
4.1	2.51	8.36	83.8	279	0.41	1.36
4.2	7.22	24.1	44.2	147	1.23	4.11
